# Repeated Intra-Arterial Thrombectomy within 72 Hours in a Patient with a Clear Contraindication for Intravenous Thrombolysis

**DOI:** 10.1155/2015/872817

**Published:** 2015-01-27

**Authors:** Mona Laible, Markus Möhlenbruch, Werner Hacke, Martin Bendszus, Peter Arthur Ringleb, Timolaos Rizos

**Affiliations:** ^1^Department of Neurology, University Hospital Heidelberg, 69120 Heidelberg, Germany; ^2^Department of Neuroradiology, University Hospital Heidelberg, 69120 Heidelberg, Germany

## Abstract

*Introduction*. Treating patients with acute ischemic stroke, proximal arterial vessel occlusion, and absolute contraindication for administering intravenous recombinant tissue plasminogen activator (rtPA) poses a therapeutic challenge. Intra-arterial thrombectomy constitutes an alternative treatment option. *Materials and Methods*. We report a case of a 57-year-old patient with concomitant gastric adenocarcinoma, who received three intra-arterial thrombectomies in 72 hours due to repeated occlusion of the left medial cerebral artery (MCA). *Findings*. Intra-arterial recanalization of the left medial cerebral artery was performed three times with initially good success. However, two days later, the right medial cerebral artery became occluded. Owing to the overall poor prognosis at that time and knowing the wishes of the patient, we decided not to perform another intra-arterial recanalization procedure. *Conclusion*. To our knowledge, this is the first case illustrating the use of repeated intra-arterial recanalization in early reocclusion of intracranial vessels.

## 1. Introduction

Evidence-based therapeutic options for patients with acute cerebral ischemia and an absolute contraindication for intravenous thrombolysis are highly limited. Although intra-arterial thrombectomy (IT) represents an alternative approach to revascularizing particular proximal intracerebral artery occlusions, no data exist concerning repeatedly performed IT in patients with early reocclusion after initial IT, to our knowledge. Here, we report the case of a patient who received three intra-arterial thrombectomies in 72 hours due to repeated occlusion of the left medial cerebral artery (MCA).

## 2. Case Report

**Figure 1:**
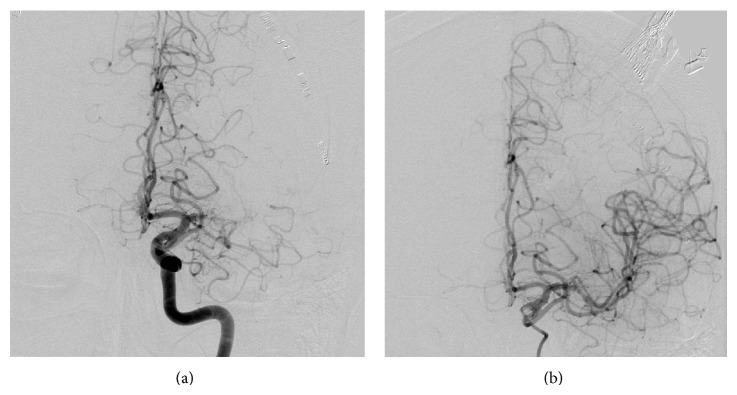
Findings before (a) and after (b) the first thrombectomy with 5 thrombectomy maneuvers of the left medial cerebral artery in the M1 segment.

A 57-year-old, nonsmoking man with newly diagnosed gastric adenocarcinoma (cT3 cN+cM1) was transferred to our department because of the sudden onset of right hemiparesis and global aphasia. Except for the gastric carcinoma, the patient was previously healthy with no medical history of cerebrovascular risk factors. Initial cerebral computed tomography (cCT) was normal. CT angiography (CTA) revealed an occlusion of the left MCA (M1 segment) without relevant arteriosclerosis of the carotid arteries. Due to active systemic bleeding with severe melena as a result of his tumor, intravenous thrombolysis was contraindicated. Therefore, we decided to conduct mechanical thrombectomy, which was successful after five thrombectomy maneuvers and intra-arterial administration of 10 mg recombinant tissue plasminogen activator (rtPA; Figure 1, TICI III; symptom-to-recanalization time: 90 min). Clinical symptoms improved remarkably (National Institutes of Health Stroke Scale, NIHSS 21 to 3) until the next day. Then, aphasia and hemiparesis worsened and CTA revealed recurrent occlusion of the left MCA (M2 segment). Again, intra-arterial thrombectomy was conducted. One thrombectomy maneuver resulted in recanalization and considerable clinical improvement (TICI III, symptom-to-recanalization time 110 min; NIHSS 11 to 3). Two days later, hemiparesis developed again on the right side and global aphasia also recurred; NIHSS worsened from 2 to 10 points. At this time, magnetic resonance imaging (MRI) again detected occlusion of the middle branch of the left MCA in the M2 segment and revealed a relevant diffusion/perfusion mismatch. Once again, thrombectomy was conducted, but the clinical result was less satisfactory (TICI IIb, symptom-to-recanalization time 240 min, NIHSS 10 to 9). Less than 5 hours after the last thrombectomy, the patient suffered a general epileptic seizure. Clinical stroke severity was unchanged (NIHSS 10). Another MRI displayed a larger diffusion weighted imaging (DWI) lesion in the left MCA territory without vessel occlusion or mismatch on diffusion/perfusion weighted imaging (PWI).

Another two days later, the clinical state of our patient deteriorated further (NIHSS 16). Radiographic findings (CCT and CTA) at this time revealed an occlusion of the right MCA (M1 segment). Due to the overall poor prognosis at that time and knowing the wishes of the patient, we decided not to perform another IT. The patient died five days later.

## 3. Discussion

To our knowledge, this is the first report of repeated intra-arterial thrombectomy in acute ischemic stroke. While no corresponding vessel stenosis was present in the MCA or carotid artery and all other diagnostic tests, including continuous electrocardiogram (ECG) monitoring, were normal, we assume that a prothrombotic state caused by the malignancy was the reason for the repeated arterial occlusions, as strokes are very common in the presence of a malignancy [1, 2]. Particularly acute stroke patients with adenocarcinomas face a substantial short-term risk of recurrent ischemic stroke [3] and an autopsy study of patients with different malignancies reported cardiovascular disease with thromboembolism in up to 15% of patients [4].

No intraprocedural complication developed in our patient, but he finally died due to repeated severe cerebral thromboemboli. In our patient, antiplatelet therapy was initiated for secondary stroke prevention. However, the optimal secondary stroke prevention in this particular group of patients remains unknown. Randomized trials comparing anticoagulation to antiplatelet therapy in patients with cancer and first-ever acute ischemic stroke may contribute to this important clinical question (e.g., clinicaltrials.gov NCT01763606).

Whereas the efficacy of intravenous rtPA has been proven in multiple randomized controlled trials [5], no evidence-based treatment alternatives exist for patients with a clear contraindication for rtPA. The three larger multicenter studies of intra-arterial thrombectomy in patients with an occlusion of the first segment of the MCA, the ICA, or basilar artery did not show that IT was superior to intravenous thrombolysis [6–8]. Our case demonstrates impressively that even repeated intracranial intra-arterial thrombectomy is technically feasible and safe and can induce a remarkable clinical improvement. However, the value of IT in patients with a contraindication to intravenous thrombolysis was not the subject of the aforementioned studies.

## 4. Conclusion

Repeated intra-arterial thrombectomy within a short time is technically feasible and was safe in our patient. Further research, particularly in the relevant subgroup of patients in whom alternative treatment would consist of purely conservative, observant, and standard stroke treatment, is urgently needed.
